# Ecogenomics and cultivation reveal distinctive viral-bacterial communities in the surface microlayer of a Baltic Sea slick

**DOI:** 10.1038/s43705-023-00307-8

**Published:** 2023-09-18

**Authors:** Janina Rahlff, Matthias Wietz, Helge-Ansgar Giebel, Oliver Bayfield, Emelie Nilsson, Kristofer Bergström, Kristopher Kieft, Karthik Anantharaman, Mariana Ribas-Ribas, Hannah D. Schweitzer, Oliver Wurl, Matthias Hoetzinger, Alfred Antson, Karin Holmfeldt

**Affiliations:** 1 Centre for Ecology and Evolution in Microbial Model Systems (EEMiS), Department of Biology and Environmental Science, Linnaeus University, Kalmar, Sweden; 2 Alfred Wegener Institute Helmholtz Centre for Polar and Marine Research, Bremerhaven, Germany; 3 Max Planck Institute for Marine Microbiology, Bremen, Germany; 4 Institute for Chemistry and Biology of the Marine Environment (ICBM), Carl von Ossietzky University of Oldenburg, Oldenburg, Germany; 5 York Structural Biology Laboratory, Department of Chemistry, University of York, York, UK; 6 Department of Bacteriology, University of Wisconsin-Madison, Madison, WI USA; 7 Center of Marine Sensors (ZfMarS), Institute for Chemistry and Biology of the Marine Environment (ICBM), Carl von Ossietzky University of Oldenburg, Wilhelmshaven, Germany; 8 The Arctic University of Norway, Tromsø, Norway; 9 Present Address: Center for Marine Sensors (ZfMarS), Institute for Chemistry and Biology of the Marine Environment (ICBM), Carl von Ossietzky University of Oldenburg, Wilhelmshaven, Germany

**Keywords:** Microbial ecology, Water microbiology, Microbial ecology, Biogeochemistry, Next-generation sequencing

## Abstract

Visible surface films, termed slicks, can extensively cover freshwater and marine ecosystems, with coastal regions being particularly susceptible to their presence. The sea-surface microlayer (SML), the upper 1-mm at the air-water interface in slicks (herein slick SML) harbors a distinctive bacterial community, but generally little is known about SML viruses. Using flow cytometry, metagenomics, and cultivation, we characterized viruses and bacteria in a brackish slick SML in comparison to non-slick SML as well as seawater below slick and non-slick areas (subsurface water = SSW). Size-fractionated filtration of all samples distinguished viral attachment to hosts and particles. The slick SML contained higher abundances of virus-like particles, prokaryotic cells, and dissolved organic carbon compared to non-slick SML and SSW. The community of 428 viral operational taxonomic units (vOTUs), 426 predicted as lytic, distinctly differed across all size fractions in the slick SML compared to non-slick SML and SSW. Specific metabolic profiles of bacterial metagenome-assembled genomes and isolates in the slick SML included a prevalence of genes encoding motility and carbohydrate-active enzymes (CAZymes). Several vOTUs were enriched in slick SML, and many virus variants were associated with particles. Nine vOTUs were only found in slick SML, six of them being targeted by slick SML-specific clustered-regularly interspaced short palindromic repeats (CRISPR) spacers likely originating from Gammaproteobacteria. Moreover, isolation of three previously unknown lytic phages for *Alishewanella* sp. and *Pseudoalteromonas tunicata*, abundant and actively replicating slick SML bacteria, suggests that viral activity in slicks contributes to biogeochemical cycling in coastal ecosystems.

## Introduction

**Fig. 1:**
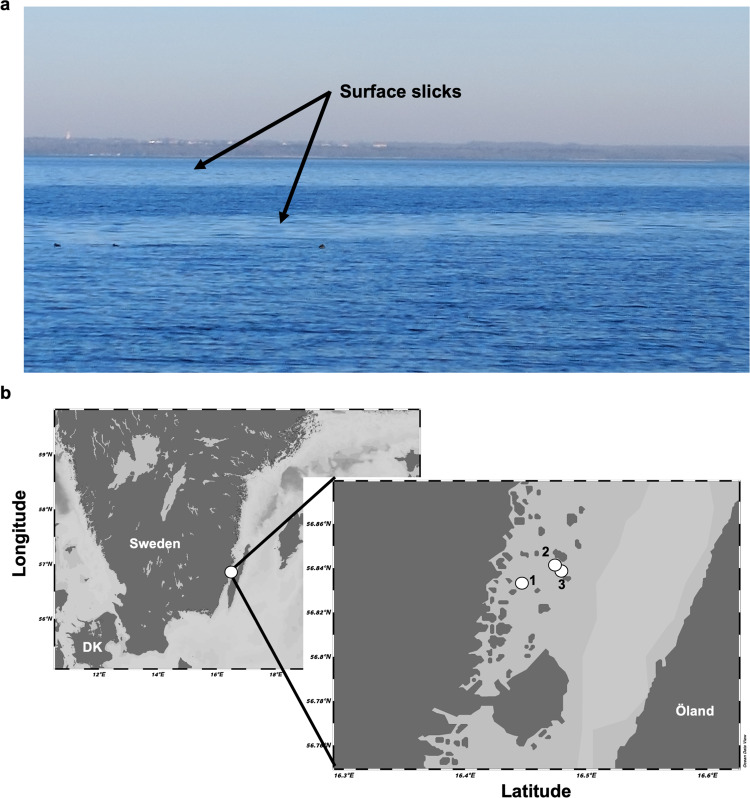
Slicks in the marine environment and sampling sites for this study. Representative example of surface slicks (none of the ones sampled), observed in the Kalmar Sound with Öland in the background (**a**). Map illustrating slick sampling sites 1, 2, and 3 close to Ljungnäs (Rockneby, Sweden) in the Baltic Sea. Map was generated using Ocean Data View v.5.6.2 [128] (**b**).

The air-sea interface spans 70% of Earth’s surface area and contains a diverse microbial community referred to as neuston [1], globally constituting 2 × 10^23^ cells [2]. In the uppermost 1-mm of the oceanic water column, termed the sea-surface microlayer (SML), the inhabiting organisms and viruses encounter highly dynamic conditions. The SML has been considered an “extreme” habitat influenced by high solar radiation, wind-wave interaction, accumulation of pollutants, sudden changes in temperature and salinity, and contact to rainfall and atmospheric depositions, among other parameters [3–8]. While the abundance, diversity and function of eukaryotes, bacteria, and archaea [9–12] have been studied in the SML, little is known about residing viruses (reviewed by Rahlff [13]). This is particularly true for the SML within natural surface slicks. Surface slicks form during low wind speeds and represent areas of accumulating surfactants, which by dampening small-scale capillary waves enhance formation of a coherent surface film (Fig. 1a [14, 15]). Surface slicks are widely distributed, with greater prevalence in coastal regions compared to the open ocean (covering on average 30 versus 11% of surface area), but can occasionally cover the surface to up to 80% in coastal waters [16]. Slicks are often enriched in cyanobacteria such as *Trichodesmium* [17–19], and in their presence, a decrease in salinity with a concurrent increase in temperature of the surface slick water has been reported [20], indicating a suppression of evaporation. Slicks also function as nurseries and dispersal agents for higher trophic levels [21, 22], plus having an important function in the suppression of air-sea carbon dioxide fluxes [23, 24]. Despite being little understood as microbial habitats to date (reviewed by Voskuhl and Rahlff [25]), slicks can accumulate and spread bacteria [26], and the bacterial community of slick SML remarkably differs from that of non-slick SML probably due to biofilm-like properties [27]. Based on 16S rRNA fingerprinting, Stolle et al. [28] reported different particle-associated and free-living bacterial communities in the Baltic Sea during formation and disintegration of a surface slick, with strong changes among free-living bacterial communities during slick disintegration. Other studies reported the presence of surfactant-producing bacteria like *Bacillus* and *Pseudomonas* spp. in and under the SML of natural surface slicks [29–31].

Investigations on viruses reported increased virus-mediated mortality, increased virus-like particle (VLP) abundance, and higher virus-host ratios in non-slick SML compared to underlying water [32, 33]. Work on Lake Baikal described autochthonous bacteriophage communities in the microlayer [34], but such studies are lacking for marine systems. A pressing question in virioneuston research is whether viruses respond to harsh environmental conditions at the air-sea boundary by lysogeny, or if high host abundances favor lytic infections according to the “kill-the-winner” model, where viruses periodically decimate the most abundant hosts [35, 36]. There is evidence for both, i.e. predominant lytic activity [37] as well as the prevalence of prophages [38] in the SML compared to underlying water. In addition, viral abundance and diversity in surface slicks are unknown, and a comparison to non-slick SML is missing. Slicks often accumulate foams [27], and increased VLP numbers have been shown for particle-enriched sea foams floating on the SML [33]. Furthermore, slick SML is enriched with transparent exopolymer particles [27] that can absorb viruses [39], but to which extent viruses, particularly bacteriophages, are associated with particles within slicks remains a knowledge gap.

The SML is a net heterotrophic system [40], as primary producers suffer from photoinhibition [41]. During the “viral shunt” [42] when viruses lyse their hosts resulting in the release of organic matter, heterotrophy might be further supported in the SML. By contributing to the release of surfactants and organic matter, the virioneuston could effectively feed the biological carbon pump, e.g., by facilitating organic particle formation and subsequent export to the seafloor (“the viral shuttle”, reviewed by [43]). Furthermore, lowering surface tension can facilitate the transfer of organic matter between atmosphere and hydrosphere.

In this work, we combined metagenomics, flow cytometry, and cultivation to reveal viral-bacterial dynamics in the SML of a surface slick from the coastal Baltic Sea compared to non-slick SML and underlying water. We accounted for the role of virus-host and virus-particle associations via size-fractionated filtration of water samples. Furthermore, patterns in the clustered-regularly interspaced short palindromic repeats (CRISPR)-Cas systems—the adaptive immune systems of bacteria—revealed past infection histories within the SML microbiome.

## Materials and methods

### Sampling

Water samples from three sites (#1 – #3) inside and outside a surface slick were collected from a small boat in Kalmar Sound, at Ljungnäs near Rockneby, Sweden on the 31st of May 2021 (Fig. 1b and Table S1). The SML from slick and non-slick areas was collected using the glass plate method [44], a suitable method to collect virioneuston [32, 37, 45]. The glass plate was rinsed with 70% ethanol followed by ambient water before sampling. Glass plate sampling is performed by immersing the custom-made plate (dimension: 30 × 40 cm, thickness: 6 mm) perpendicularly to the ocean surface and slowly withdrawing it vertically at a speed of 5–6 cm s^−1^ [46]. Sampled SML is scraped from both sides of the plate with a silicone rubber squeegee blade and collected via a funnel in bottles (Fig. S1). Since this method is for small volumes, the glass plate dipping was repeated several times until a volume of ~800 ml was sampled. Reference water (subsurface water = SSW) was collected from ~70 cm depth below the slick and non-slick area with a Hydro-Bios 1-l Ruttner water sampler (Swedaq, Höör, Sweden, Fig. S1). Wind speed was measured with a hand-held anemometer model MS6252A (Mastech Group, Brea, CA, USA). Site #1 was sampled for metagenomics, dissolved organic carbon (DOC), surfactants, and bacterial isolation, while sites #1 – #3 were additionally sampled for flow cytometry and phage isolation (Table S1). For DNA extraction (see below), water samples (600–700 and 2100 ml of SML and SSW, respectively) were sequentially filtered through polycarbonate filters with 5 µm and 0.2 µm pore size (Nucleopore Track-Edged Membrane, Whatman, Maidstone, UK) to obtain the particle-associated (PA) and free-living (FL) fraction, respectively. The PA fraction contains all organisms >5 µm size including phytoplankton, protists, virions attached to particles or hosts as well as intracellular viruses. Microbes between 5 and 0.2 µm size include host-associated viruses in the FL fraction. The flow-through of the 0.2 µm pore size filtration was chemically flocculated [47] using a higher iron-III-chloride concentration (final concentration: 10 mg FeCl_3_ l^−1^) than in the original protocol as recently suggested for freshwater [48], and filtered onto 1 µm pore size polycarbonate membrane filters (142 mm diameter, Whatman/GE Healthcare, Uppsala, Sweden) to obtain the viral fraction. All filter membranes were stored at −80 °C until DNA extraction.

### Surfactant and DOC analysis

Surfactant concentration was measured by the voltammetry 747 VA Stand (Metrohm, Herisau, Switzerland) with a hanging mercury drop electrode. Surfactants accumulate at the mercury drop at a potential of −0.6 V /versus an Ag/AgCl reference electrode. Surfactants were quantified in 10 ml of unfiltered samples with the standard addition technique with details given in [49]. For DOC, duplicates of 30 ml sample water and a MilliQ control were gravity-filtered onto precombusted (475 °C, 3 h) GF/C glass fiber filters (nominal pore size ~1.2 µm), acidified (200 µl 2 M HCl), and stored in precombusted glass vials (475 °C, 3 h) with acid-washed lids at 4 °C until analysis as described previously [50].

### Cell count and VLP measurements

Unfiltered slick SML, non-slick SML, slick SSW and non-slick SSW from sites #1 – #3 (Fig. 1b) were fixed with 25% glutardialdehyde (0.5% final concentration, Sigma-Aldrich/Merck Life Science AB, Solna, Sweden) and stored at −80 °C. Samples experienced an extra freeze-thaw cycle during shipment. While comparisons with other studies might be difficult, we are confident that comparisons between samples treated the same are reasonable. Prokaryotic cells and VLPs were measured on a flow cytometer (BD Accuri C6, BD Biosciences, Franklin Lakes, NJ, USA) by using protocols from [51] and [52], respectively. Enrichment factors (EF) were calculated for flow cytometry data and metagenome coverages of viral OTUs (see below) as a ratio of a factor in the SML divided by the SSW counterpart, with EF > 1 and EF < 1 indicating enrichment and depletion in the SML, respectively.

### Isolation of bacteria

Bacteria were isolated from the slick SML of site #1 by plating 100 µl undiluted and diluted (10^−1^–10^−4^) sample on Zobell Agar (1 g yeast extract (BD), 5 g bacto-peptone (BD), 15 g bacto agar (BD), 800 ml Baltic Sea water, 200 ml Milli-Q water). Plates were incubated at room temperature (~22 °C). Bacteria of different color and morphology assumed to represent different species were pure-cultured from single colonies thrice before they were inoculated in Zobell medium (1 g yeast extract (BD), 5 g bacto-peptone (BD), 800 ml Baltic Sea water, 200 ml Milli-Q water) over night, and stored as glycerol stocks (600 µl 50% glycerol (Sigma) and 900 µl bacterial culture) at −80 °C.

### Phage isolation and plaque assay

Water from all sampling sites was filtered through a 0.2 µm pore size PES syringe filter and the flow-through collected for phage isolation using plaque assay following Nilsson et al. [53]. Briefly, 500 µl of the water sample was mixed with 3.5 ml top agar (450 mM NaCl (Sigma), 50 mM MgSO_4_ x 7 H_2_O (Sigma), 50 mM Trizma base (Sigma), 5 g l^−1^ low-melting agarose (Thermo Scientific, Waltham, MA, USA)) and 300 µl overnight bacterial culture of *Alishewanella* sp. SMS8 or *Pseudoalteromonas tunicata* SMS2 (Table S2a). Plates were incubated on the bench overnight, and plaque-forming units were monitored over 48 h. Plaques of different size and morphologies were picked from plates using a sterile 100 µl pipet tip and stored in MSM buffer ( = top-agar without low-melting agarose) at 4 °C. Phages were purified by replating thrice before two fully lysed plates per viruses were harvested with 5 ml MSM buffer. The phage-MSM mixture was centrifuged at 3260 × g for 20 min, and the supernatant was filtered through a 0.2 µm pore size syringe filter and stored at 4 °C. The phages were stored both as free phages at 4 °C and in infected hosts at −80 °C. For infected hosts, the 400 µl freshly harvested phage stock was mixed with 1.2 ml overnight bacterial culture for 15 min before being mixed with glycerol and frozen as described above.

### Transmission Electron Microscopy (TEM) imaging

TEM was conducted using high titer phage lysate and negative staining as in [54]. Briefly, phages were loaded on pre-discharged copper grids (200 Mesh Cu. Agar Scientific Ltd., Stansted, UK), stained with 2% w/v uranyl acetate (Agar Scientific Ltd), and imaged using a Tecnai 12 G2 BioTWIN microscope (FEI Company, Hillsboro, OR, USA). Phage head and tail diameter were measured with ImageJ v.1.53 [55] according to [56].

### DNA extraction and sequencing of bacterial and phage isolates and metagenomes

DNA from 1 ml of harvested phage stock was extracted using Wizard PCR DNA Purification Resin and Minicolumns (both Promega, Madison, WI, USA) as described previously [53]. Bacterial genomic DNA from selected strains (Table S2a) was extracted using the E.Z.N.A Tissue DNA kit (Omega Bio-tek, Norcross, GA, USA) according to the manufacturer’s protocol. DNA for metagenomes was extracted from 5 and 0.2 µm pore size filters (47 mm diameter) using the DNAeasy Power Soil Pro kit (Qiagen, Sweden). DNA from viral fraction (142 mm diameter membranes) was extracted using the DNAeasy PowerMax Soil kit (Qiagen, Sweden) with a subsequent ethanol precipitation step for concentrating DNA. DNA concentrations were measured on a Nanodrop 2000 spectrophotometer (Thermo Scientific) and Qubit® 2.0 Fluorometer (Invitrogen/ Life Technologies Corporation, Carlsbad, CA; USA). Sequencing was conducted by SciLifeLab (Solna, Sweden) using the Illumina DNA PCR-free Prep kit for library preparation. Samples were sequenced on NovaSeq6000 (NovaSeq Control Software 1.7.5/RTA v3.4.4) with a 151nt (Read1) – 10nt (Index1) − 10nt (Index2) − 151nt (Read2) setup using ‘NovaSeqXp’ workflow in ‘S4’ mode flowcell. The Bcl to FastQ conversion was performed using bcl2fastq_v2.20.0.422 from the CASAVA software suite. The quality scale used is Sanger / phred33 / Illumina 1.8 + . One bacterial (SMS8) and one viral (vB_PtuP_Slicky01) genome were sequenced at Eurofins Genomics using the INVIEW Resequencing bacteria (eurofinsgenomics.eu) product and the same sequencer as above. All reads went through adapter trimming and quality control using bbduk as part of BBTools [57] with settings k = 23 mink=11 hdist=1 tbo tpe ktrim=r ftm=5 with subsequent contaminant filtering using the Illumina PhiX spike-in reference genome (phix174_ill.ref.fa) and the artificial contaminants file (sequencing_artifacts.fa) from BBMap resources. Sickle v.1.33 [58] was run with pe mode and -t sanger setting. Genomes from isolates were assembled using MEGAHIT v.1.2.9 for phages [59] and SPAdes v.3.15.3 with option --isolate for bacteria [60]. Quality checks and taxonomy assignments were performed as for metagenome-assembled genomes (MAGs), see below. For genomes of bacterial isolates, we annotated several functional traits (Table S2a–g) as follows: KEGG annotations were done using KAAS [61], and pathways reconstructed using KEGG Mapper [62]. Carbohydrate-active enzymes (CAZymes) were predicted using dbCAN2 [63] only considering hits with > 60% query coverage and e-value < 1e−15. Analyses were done and visualized in R v.4.2.2 using packages tidyverse [64] and data.table [65]. Biosynthetic gene clusters were predicted using antiSMASH v.6.1.1 [66]. Genes involved in surfactant biosynthesis (NCBI accessions AAD04757.1, AEW31038.1, NP_252169.1, PBL99399.1, BAG28347.1, AAB35246.1) were searched using Custom-BLAST in Geneious Prime [67]. For comparison, the lichenysin gene cluster was obtained from https://mibig.secondarymetabolites.org [68].

### Binning and functional analysis of bacterial genomes

Taxonomic profiling of bacteria was conducted with mOTUs v.3.0.2 [69, 70]. using trimmed reads with options -A (reports full taxonomy) -c (reports counts) -M (to save intermediate marker gene cluster count) and a separate run to retrieve unassigned taxa. The tool mOTUs employs phylogenetic marker gene sequences that are universal, protein-coding, and single-copy to evaluate the taxonomic composition of microbial communities derived from metagenomes [69, 70]. Resulting read counts were read-sum normalized, and Shannon-Wiener index and relative abundance for beta-diversity (also for the viral clusters (VC), see below) were investigated using phyloseq package [71] in the R programming environment [72]. Binning of MAGs was performed using CONCOCT v.1.1.0 [73] and MetaBAT v.2.12.1 [74] on MetaSPAdes [60] v.3.15.3 assemblies previously filtered to a minimum length of 1000 bps. A non-redundant set of bins was created with DAS_Tool v.1.1.3 [75] with default score threshold and followed by manual refinement in uBin v.0.9.14 [76] using information on GC content, coverage and taxonomy. MAGs underwent quality checks in CheckM v.1.1.3 [77], followed by taxonomic classification with the classify_wf option in GTDB-Tk v.1.7.0. and database version r202 [78]. MAGs were used for further analysis if they reached estimated completeness and contamination scores of ≥70% and ≤10% in either uBin or CheckM. Mapping to MAGs and isolate genomes was performed with Bowtie v.2.3.5.1 [79] using the --reorder flag. Mismatch filtering with 2% error rate (-mm 3) was conducted within iRep v.1.1.0, which was used to estimate in situ replication rates at default thresholds [80]. Average nucleotide identity (ANI) comparison was carried out using FastANI v.1.33 [81] and visualized in ANIclustermap (https://github.com/moshi4/ANIclustermap). KEGG annotations derived from predictions with DRAM v.1.2.4 [82] were compared for significant differences between MAG groups using ALDEx2 [83]. The number of hits were normalized by the number of genes per MAG. CAZymes were predicted as mentioned above for bacterial isolates.

### Metagenomic analyses of viruses

Trimmed reads were assembled twice using MetaSPAdes v3.15.3 [60] and the Metaviral SPAdes [84] option. For viral analysis, assemblies were combined, filtered to keep scaffolds of minimum 1 kb, and viruses were identified using VIBRANT v.1.2.1 [85] by adding the --virome option for the viral fractions, and VirSorter v.2 with --include-groups “dsDNAphage,ssDNA” and default score [86]. The output was combined and filtered to 10 kb sequence length. Only viruses with attributes “medium quality”, “high quality” or “complete” determined using CheckV v.0.8.1 were used for downstream analyses. VIRIDIC v1.1 [87] was run for genus and species clustering, and only one representative of a viral species cluster (preferably a circular or the longest scaffold of the cluster) was used for further analysis. This workflow resulted in 428 representative viral scaffolds (further referred to as viral operational taxonomic units = vOTUs) as the final output. Viral relative abundance (depth of coverage) and breadth of coverage for vOTUs and phage isolates (see below) was calculated with the calc_coverage_v3.rb (https://github.com/ProbstLab/uBin-helperscripts/blob/master/bin/04_01calc_coverage_v3.rb) [76] and the calcopo.rb script (https://github.com/ProbstLab/viromics/blob/master/calcopo/calcopo.rb) [88], respectively, after read-mapping with Bowtie2. We followed viromics conventions of [89], and only considered vOTUs > 10 kb length with coverage of 90% identical reads (achieved with Bowtie2 settings: --ignore-quals –mp = 1,1 –np = 1 –rdg = 0,1 –rfg = 0,1 --score-min = L,0,−0.1 [54]), and 75% of the vOTU having a coverage of at least 1× to be present in a sample. Depth of coverage to vOTUs was normalized to sequencing depth. Viral genes in vOTUs and phage isolates were predicted using Prodigal v.2.6.3 in meta mode [90] and functionally annotated using DRAM-v v.1.2.4 [91]. Viral micro-diversity was explored using inStrain v.1.7.1 [92] in profile and compare mode on files based on the mapping as mentioned above and after conversion into .bam files using SAMtools v.1.1.7 [93]. Alignments of phage isolates (based on tBLASTx) and placement in the proteomic tree were inferred from and conducted within VipTree v.3.5 [94] (version of Virus-Host database: RefSeq release 217) by selecting dsDNA as nucleic acid type and defining a subset of the closest phylogenetic representatives. Clustering of vOTUs with reference database phages (release July 2022, from https://github.com/RyanCook94/inphared) [95] was performed using vConTACT2 v.0.9.19 [96], VC information was compiled using graphanalyzer v.1.5.1. (https://github.com/lazzarigioele/graphanalyzer) [97], and the network visualized using Cytoscape v.3.9 [98]. Viral taxonomic information was obtained from PhaGCN2.0 [99]. Auxiliary metabolic genes (AMGs) on vOTUs were detected using annoVIBRANT (https://github.com/AnantharamanLab/annoVIBRANT), using modified scripts of VIBRANT v.1.2.1 [85] to report AMGs of vOTUs. For this analysis, a vOTU carrying an AMG was attributed to a sample if present based on read mapping conventions [89], followed by a calculation of the sum of coverages of phages carrying that AMG. Comparisons of enrichments of a vOTU inside and outside the slick was done by calculating coverage ratios, i.e., the coverage of a virus in slick SML divided by the coverage in slick SSW and the same procedure for the non-slick vOTUs. EF was calculated for coverage of VCs as explained above.

### CRISPR analysis and virus-host matches

Viral OTUs were matched to a set of MAGs previously dereplicated with dRep v.3.4.0 with default parameters [100] using VirHostMatcher v.1.0.0 [101] and a d2* dissimilarity threshold of 0.3. Prophages in dereplicated MAGs and isolate genomes were detected with VIBRANT. CRISPRcasFinder v.4.2.20 [102] was used to detect CRISPR arrays in MetaSPAdes assemblies (>1 kb), MAGs, and genomes of bacterial isolates. CRISPR direct repeat (DR) sequences from assemblies were extracted from evidence level 4 CRISPR systems (Table S3a). DR sequences were blasted against vOTUs, and DRs with a hit at 100% similarity were deleted to avoid extraction of false-positive spacers from vOTUs. Remaining DRs (Table S3b) were fed into MetaCRAST [103] to extract spacer sequences from raw reads using settings -d 3 -l 60 -c 0.99 -a 0.99 -r. Spacers were subsequently filtered for homopolymers and length (20–60 bp), only considering CRISPR spacer to viral protospacers matches with 100% similarity (very strict filtering due to high amount of matches). Spacer-protospacer matches between MAGs, bacterial isolates, and viruses were filtered at 80% similarity.

## Results

### Surfactants, DOC, VLPs, and prokaryotic cells are enriched in the slick SML

**Fig. 2:**
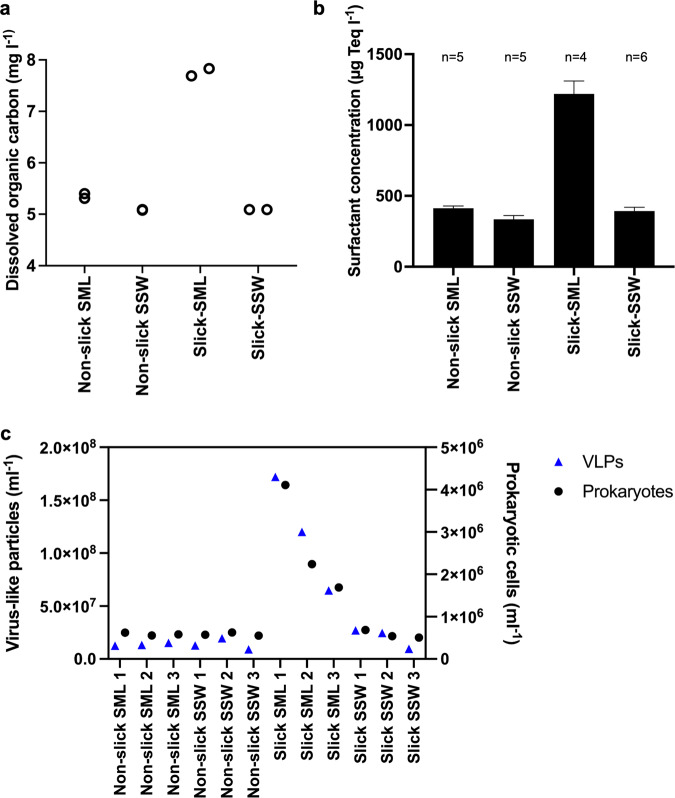
Accumulation of organic matter, surfactants, prokaryotic cells, and virus-like particles (VLPs) in water samples. Dissolved organic carbon (DOC) measured in technical duplicates (**a**), mean +/- standard deviation for concentration of surfactants (n = number of technical replicates) (**b**), and counts of VLPs and prokaryotic cells in slick SML, non-slick SML, slick SSW and non-slick SSW (**c**). DOC and surfactants were measured from sampling site #1 only. SML sea-surface microlayer, SSW subsurface water (~70 cm depth).

Slick SML was enriched in DOC (7.69 and 7.83 mg l^−1^) in comparison to non-slick SML (5.31 and 5.40 mg l^−1^) and both SSW samples (5.08 and 5.09 mg l^−1^) (Fig. 2a). The same pattern was seen for surfactants in slick SML (mean = 1219.3 µg Triton-X-100 equivalent (Teq) l^−1^) compared to the other three sample types (334.3–412.1 µg Teq l^−1^, Fig. 2b). Prokaryotes and VLPs from three individual surface slicks showed highest counts in the slick SML compared to non-slick SML and SSW (Fig. 2c). Prokaryotic abundance in slick SML was 2.7 × 10^6^ ± 1.3 × 10^6^ cells ml^−1^ compared to 5.9 × 10^5^ ± 3.5 × 10^4^, 5.8 × 10^5^ ± 9.5 × 10^4^, and 5.8 × 10^5^ ± 3.7 × 10^4^ cells ml^−1^ in the non-slick SML and SSW samples, respectively. Abundances of VLPs in slick SML was 1.2 × 10^8^ ± 5.4 × 10^7^ compared to 1.4 × 10^7^ ± 9.5 × 10^6^, 2.0 × 10^7^ ± 9.5 × 10^6^, and 1.4 × 10^7^ ± 5.3 × 10^6^ VLPs ml^−1^ in the other samples (Fig. 2c). Virus-host ratios were highest in slick SML (44.6 ± 8.0), followed by slick SSW (34.5 ± 14.0), non-slick SML (23.4 ± 3.0) and non-slick SSW (23.3 ± 7.5). Mean EFs were 6.0 ± 1.0 and 1.1 ± 0.5 for VLPs in slick SML and non-slick SML, respectively, and 4.5 ± 1.4 and 1.0 ± 0.1 for prokaryotic cells for slick SML and non-slick SML, respectively.

### Higher bacterial diversity in slick SML, with Gammaproteobacteria as dominant class

**Fig. 3:**
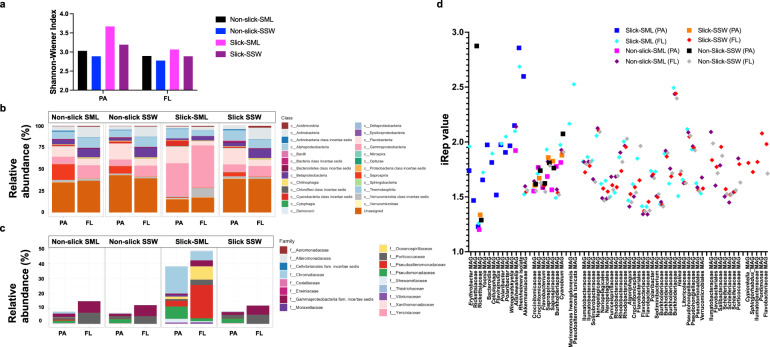
Diversity and indices of replication (iRep) for slick-associated bacteria. Shannon-Wiener Index for PA and FL bacterial fractions from slick versus non-slick samples (**a**), relative abundance of bacterial classes among PA and FL fractions (**b**), and families of Gammaproteobacteria in greater detail (**c**). In situ replication rates (based on iRep) for bacterial metagenome-assembled genomes (**d**). FL free-living fraction (5–0.2 µm), PA particle-associated fraction (>5 µm), SML sea-surface microlayer, SSW subsurface water (~70 cm depth).

α-diversity was highest in the PA bacterial fraction (>5 µm) within the slick SML, illustrated by maximum Shannon-Wiener diversity index 3.7 compared to non-slick SML (3.0), slick SSW (3.2) and non-slick SSW (2.9). A similar, but weaker, trend was observed for the FL bacterial fraction (5–0.2 µm) with a Shannon-Wiener index of 3.1, 2.9, 2.9 and 2.8 for slick SML, non-slick SML, slick SSW, and non-slick SSW, respectively (Fig. 3a). Especially Gammaproteobacteria showed higher relative abundance in the slick SML in both the PA (38.5%) and FL (48.8%) fraction compared to other samples (7.5–15.1%, Fig. 3b, c). The most abundant gammaproteobacterial families in the slick SML FL and PA fraction were *Pseudoalteromonadaceae*, mostly *P. tunicata* (22.1% compared to 0.01% in non-slick SML) and *Chromatiaceae* (17.8% compared to 1.2% in non-slick SML, Fig. 3c, Supplementary Results), respectively. In the slick SML PA fraction, other abundant bacteria were *Polaribacter* spp. (8.3%, Fig. S2), *Nodularia spumigena* (4.2%), *P. tunicata* (3.8%), *Pseudomonas fluorescens* (2.9%), and *Shewanella baltica* (2.5%). The slick SML FL fraction featured unclassified Verrucomicrobia (10.7%) and *Marinomonas* (8.4%) species. On the other hand, unclassified *Porticoccaceae* were less abundant in the slick SML (3.6%) compared to non-slick SSW (7.3%) in the FL fraction.

### Abundant PA bacteria with in silico predicted activity occur in the slick SML

We recovered 316 MAGs and seven gammaproteobacterial isolates from slick SML samples and performed functional predictions to assess putative ecological traits. The following seven strains were sequenced: *P. tunicata* SMS2, *Alishewanella* sp. SMS8, SMS9, as well as *Rheinheimera baltica* SMS3, SMS4, SMS11, SMS12 (Table S2a). The 316 MAGs covered eight bacterial phyla (Table S4a). Four MAGs carried prophages: MAG_221 (*Flavobacteriaceae*), MAG_166 (*Rickettsiaceae*), MAG_147 (*Cypionkella* sp.), and MAG_137 (*Cyanobium* sp.), but among these four, only MAG_166 showed higher coverage (101 x) in slick SML compared to other samples (Tables S4a, S5). Analysis of in situ replication rates represented by the Index of Replication (iRep) suggested that several MAGs and the genome of SMS3 (representative genome of *R. baltica*) formed a distinct group of actively replicating bacteria in the slick SML PA fraction (Fig. 3d). An iRep > 2 in the PA fraction was found for MAGs of *Alishewanella*, *Akkermansiaceae*, and *R. baltica* SMS3 (Figs. 3d and S3), matching their high relative abundance in the PA fraction (Table S5). The iRep of *Marinomonas hwangdonensis* (2.2) and *P. tunicata* (2.5) MAGs suggested that these bacteria replicated in the FL fraction of the slick SML. *Alishewanella* MAG_01 as well as *Alishewanella* isolates SMS8 and SMS9 formed a joint ANI cluster (ANI ≥ 99.3%) (Fig. S4), and SMS8 and SMS9 are probably new species assigned to *Alishewanella* in GTDB-Tk classify workflow (Fig. S5). SMS8 carried a prophage likely with siphovirus morphology and a genome length of 50 kb (Table S2a, Fig. S6, Supplementary Results).

### Functional characterization of MAGs and isolates

**Fig. 4:**
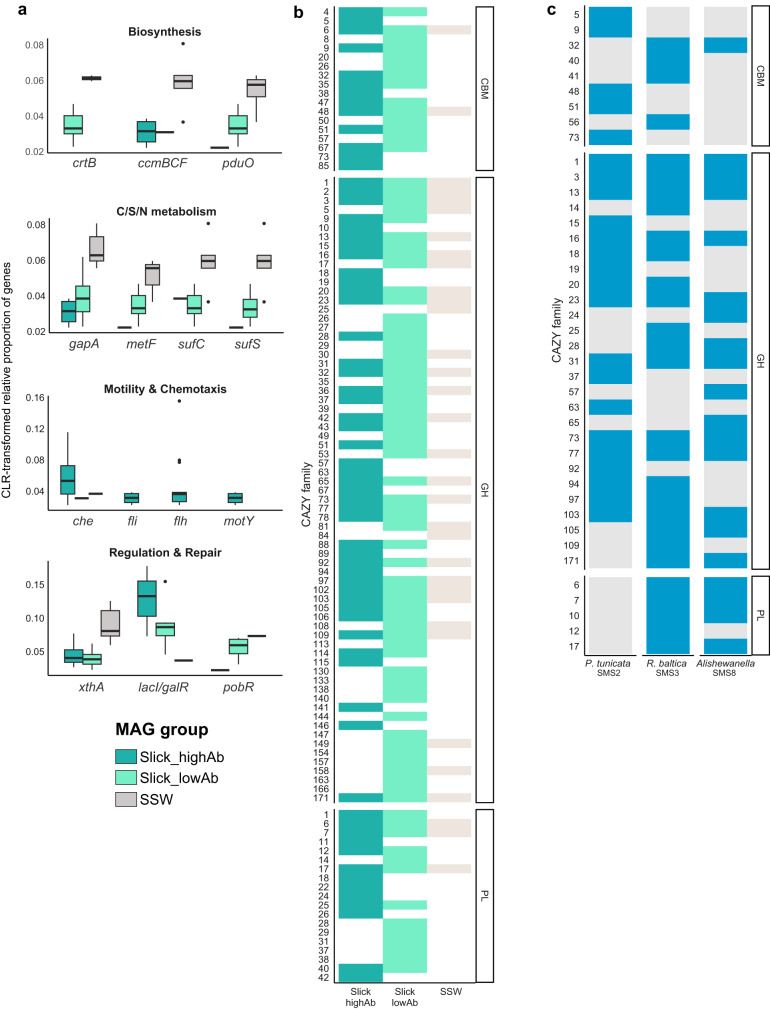
Relative fraction of genes from specific functional pathways with differential abundance between MAG groups, identified using ALDEx2 and displayed as CLR-transformed relative gene abundances. Several genes from functionally related categories (e.g. *che* chemotaxis, *fli/flh* flagellum genes) were combined, showing the average CLR value. *lacI/galR* LacI family transcriptional regulator, *xthA* exodeoxyribonuclease, *motY* sodium−type flagellar protein, *pobR* AraC family transcriptional regulator, *sufS* cysteine desulfurase/selenocysteine lyase,* sufC* Fe − S cluster assembly ATP-binding protein, *metF* methylenetetrahydrofolate reductase, *gapA* glyceraldehyde 3−phosphate dehydrogenase, *pduO* cob(I)alamin adenosyltransferase, *crtB* 15−cis−phytoene synthase, *ccmBCF* cytochrome/heme biogenesis/transport (Table S4c) (**a**). Diversity of CAZyme families in different MAG groups (see Table S4d for details) (**b**). CAZyme profiles of slick SML isolates, showing presence/absence of carbohydrate-binding module (CBM), glycoside hydrolase (GH), and polysaccharide lyase (PL) gene families (Table S2e). The four *R. baltica* isolates featured identical CAZyme diversity; therefore, only SMS3 is shown as representative. Due to lower completeness of SMS9, only SMS8 is shown for *Alishewanella* sp. (**c**).

MAGs and isolates abundant in slick SML (Table S5) were functionally analyzed by annotating KEGG modules and CAZymes, informing about central metabolic capacities and carbohydrate degradation, respectively. To identify slick- and SSW-specific features, four MAGs predominant in slick at high abundance (designated Slick_highAb), eight MAGs predominant in slick at low abundance (designated Slick_lowAb), and five MAGs predominant in the SSW (designated SSW, Table S4b) were analyzed. Approximately 250 KEGG-ids were differentially abundant between the three groups (Table S4c). For instance, flagellum genes were only found in Slick_highAb MAGs, while genes involved in regulation, repair and biosynthesis showed contrasting patterns between groups (Fig. 4a). SSW MAGs encoded more CAZymes (Fig. S7, Table S4d), with significantly higher fractions of glycoside hydrolase (two-sided Wilcoxon test, *W* = 191, *p* = 0.0040), polysaccharide lyase (*W* = 0, *p* = 0.043), and glycosyltransferase genes (*W* = 133, *p* = 0.0025). However, the diversity of CAZyme families was higher in SML-MAGs, with complementing CAZyme profiles between Slick_lowAb and Slick_highAb MAGs (Fig. 4b). Both groups of SML-MAGs encoded diverse polysaccharide lyase families, whereas the SSW group only encoded alginate-targeting PL6, PL7 and PL17 but in higher copy numbers. However, sizes of gene pools differed, with 9000 genes in SSW-MAGs compared to ~60,000 in SML-MAGs, possibly influencing these patterns.

To corroborate the distinctness of SML microbiomes, we analyzed the genomes of the seven bacterial isolates from slick SML, for which corresponding MAGs have a slick-specific high abundance and iRep compared to SSW (Tables S2a, S4b, S5). Due to low completeness of SMS9 (70.6%), we focused on SMS8. All isolates encoded gene clusters mediating chemotaxis and motility (e.g, *mcr*/*che*, *mot*, *fli*; Table S2d). *P. tunicata* SMS2 and *R. baltica* SMS3, SMS4, SMS11, and SMS12 additionally harbored a type VI secretion system involved in biofilm formation. All *R. baltica* strains featured highly similar CAZyme and KEGG profiles, with only 58 of 7850 predicted KEGG-ids not shared. We found genes encoding homoserine lactones (mediators of quorum sensing) in all *R. baltica* and in *Alishewanella* sp. SMS8 (Table S2f). SMS2 encodes a distinct CAZyme repertoire compared to other strains (Fig. 4c), indicating that SML strains specialize on different carbon sources. Most notably, SMS2 encodes no polysaccharide lyases compared to several clusters targeting alginate and pectin in the other strains (Table S2e). Instead, the unique presence of GH19 plus carbohydrate-binding module families CBM5 and CBM73 might enable chitin degradation; plus mannan, amylase and pullulan activities through GH92 and GH13 genes. CAZyme profiles of SMS2 and SMS3 differed (Fig. 4c, Table S2e), suggesting different “carbohydrate niches”. SMS2 encoded genes for violacein and prodigiosin biosynthesis (Table S2f), likely explaining its purple-blue phenotype. Checking for surfactant-related genes revealed several homologs of lichenysin and surfactin synthetase (30% amino acid identity to genes in the characterized cluster of *Bacillus licheniformis*) in strains SMS2, SMS3, and SMS4 (Table S2g).

### Viruses establish a distinctive community in the slick SML

**Fig. 5:**
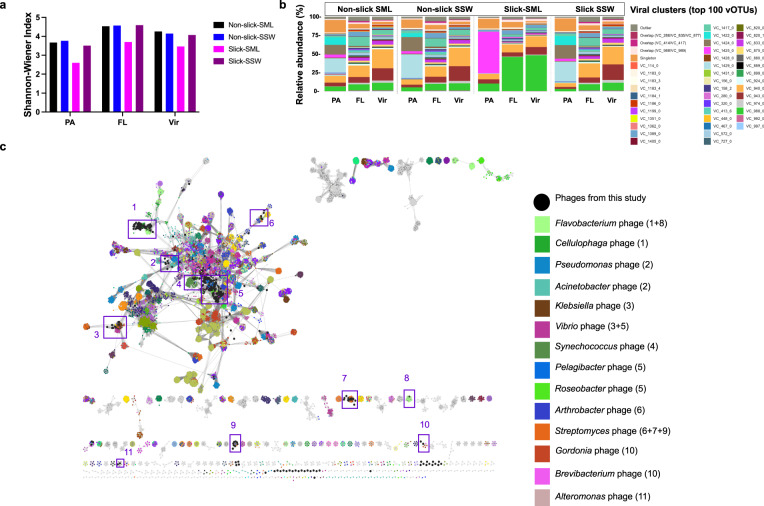
Viral diversity, relative abundance, and clustering with known phages. Shannon-Wiener Index for vOTUs for the four different sample types (**a**). Relative abundance of viral clusters (VC) including the top 100 abundant vOTUs show an increase in relative abundance of certain VC in slick SML (VC_1425_0 & VC_988_0), while other VC decreased in relative abundance (VC1424_0 or VC_572_0) compared to reference samples. Further information about VCs and closest associated viruses is given in Table S6. Outliers and singletons refer to unclustered, presumably unknown viruses (**b**). Many vOTUs (nodes) from this study clustered with known phages of *Flavobacterium*, *Pelagibacter*, or *Synechococcus* based on shared protein clusters (interactions with known phages indicated by eleven purple frames) with a virus reference database from July 2022. Several vOTUs clustered only with other vOTUs from this study indicating unknown viruses (**c**). FL free-living fraction (5–0.2 µm), PA particle-associated fraction (>5 µm), SML sea-surface microlayer, SSW subsurface water (~70 cm depth), Vir viral fraction (<0.2 µm).

Of 428 vOTUs >10 kb length (dsDNA viruses) dereplicated at the species level, 16, 45, and 367 were complete, high, or medium quality, respectively. Only two vOTUs were determined to be proviruses according to CheckV (Table S6). Different vOTUs were assembled in each sample type and size-fraction; however, based on read mapping, most vOTUs and VCs were shared between all four sample types (Figs. S8 and S9). Certain vOTUs were unique for the slick SML, while others were found in all sample types except slick SML (Fig. S8) agreeing with a correlation matrix showing that the slick SML vOTU community was most distinct from the other samples (Fig. S10), with highest coverage in the viral fractions (Fig. S11). Opposed to bacterial α-diversity, vOTUs were more diverse in the FL and viral fraction (Shannon-Wiener index: range 3.4–4.6) compared to PA (range 2.5–3.8), but among each filtered fraction diversity was always lowest in slick SML (PA = 2.6, FL = 3.7, viral = 3.5, Fig. 5a). Certain viral clusters (VCs) showed markedly higher relative abundance in slick SML, such as VC_988_0 with relative abundance of ~43% (slick SML) compared to ~8% (non-slick SML) (Fig. 5b). According to the vConTACT2 analysis, VC_988_0 shares protein clusters with Flavobacteria phages (Fig. 5c cluster 1). In the PA fraction, vOTUs of VC_1425_0 carrying a reverse transcriptase gene showed a relative abundance of 51.5% in slick SML compared to 3.3% in non-slick SML. Nine vOTUs were exclusively detected in the slick SML. Among those, the overlap cluster VC_580/VC_601, sharing genomic similarities with various VCs containing known *Shewanella* phages, contained two vOTUs (34.1 kb and 40.7 kb length) that were solely detected in slick SML PA fraction (Fig. S8g).

**Fig. 6:**
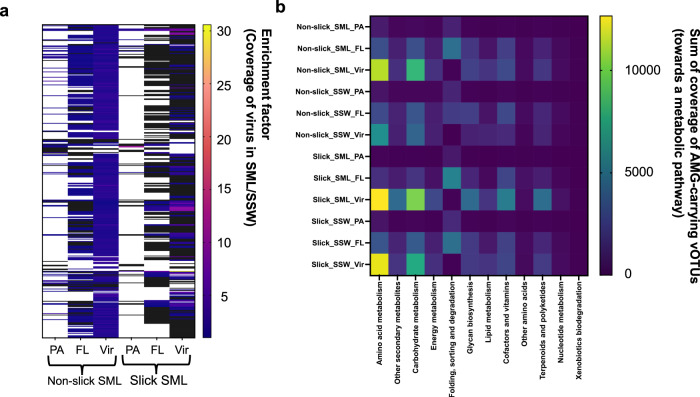
Viral enrichment in slick versus non-slick SML, and auxiliary metabolic genes. Enrichment of viruses in slick SML and non-slick SML across different filtered fractions. Shown are coverage ratios >1 indicating enrichment of a virus in the SML compared to the corresponding reference subsurface water sample. Black areas indicate depletion of a virus (ratio <1), while white areas show absence of the virus in the nominator or denominator of the ratio. Values and VCs of the heatmap’s y-axis are given in Table S8a (**a**). Coverage of vOTUs carrying an auxiliary metabolic gene (AMG), sorted by KEGG category (**b**). Only vOTUs being present in a sample based on read mapping were considered for this analysis. Full information on involved AMGs is given in Table S8b. FL free-living fraction (5–0.2 µm), PA particle-associated fraction (>5 µm), SML sea-surface microlayer, SSW subsurface water, Vir viral fraction (<0.2 µm).

Less VCs became enriched (EF > 1) in the PA and FL fraction of the slick SML compared to non-slick SML (Fig. 6a), in agreement with α-diversity (Fig. 3a). However, the few vOTUs that were enriched in the slick SML often reached very high EFs (>6), e.g., subclusters of VC_988_0, VC_975_0 (resembling *Pelagibacter* phage HTVC028P), VC_1075_0 (resembling *Gordonia* phage GMA6), VC_1182, VC_1425_0, as well as several singletons and outliers, presumably representing previously unknown viruses (Tables S6 and S7). Likely due to their higher abundance, viruses contributed to the prevalence of AMGs in the slick SML (Fig. 6b, Table S8 a, b, Fig. S12), namely genes related to amino acid metabolism (mainly arginine, proline, alanine, aspartate and glutamate metabolism), carbohydrate metabolism (amino sugar, nucleotide sugar, fructose and mannose metabolism), or to cofactors and vitamins (e.g. folate biosynthesis). Two abundant 106.6 kb and 57.4 kb vOTUs from the slick SML, both unclustered in vConTACT2, contained the gene *folA* (dihydrofolate reductase, KEGG enzyme EC:1.5.1.3), which has an essential role in DNA synthesis.

### Virus-host predictions and slick SML-specific CRISPR spacer-protospacer matches

**Fig. 7:**
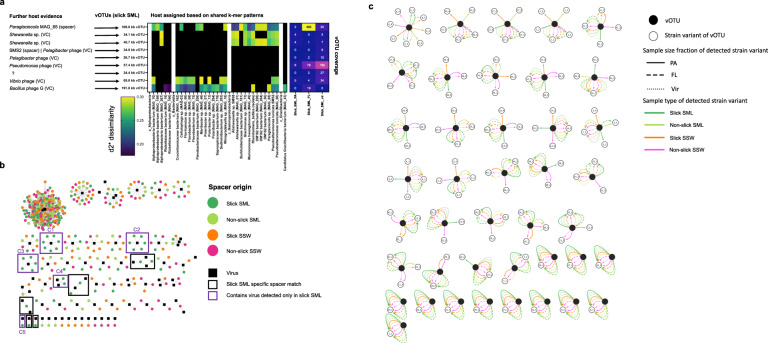
Phage-host interactions and viral micro-diversity. Based on k-mer frequency patterns, vOTUs were predicted to match host MAGs and isolate genomes (middle). Further host evidence (left) was derived from vConTACT2 viral clustering (VC) with known phages from reference database and CRISPR spacer matches from MAGs. The heatmap (right) depicts the coverage of vOTUs in the three size fractions. D2* is a dissimilarity measure (the lower, the higher the similarity) (**a**) CRISPR-spacer to vOTU protospacer matches at 100% similarity reveal ten clusters with slick SML derived spacers, with C1-C5 including a slick SML specific vOTU from (**a**), framed in purple (**b**). Viral micro-diversity for different water sample types and filtered size fractions. Open circles represent strain variants of the viral OTUs (closed circles) and lines indicate the sample in which the respective variant has been detected. This figure corresponds to the results shown in Table S12b. FL free-living fraction (5–0.2 µm), PA particle-associated fraction (>5 µm), SML sea-surface microlayer, SSW subsurface water, Vir viral fraction (<0.2 µm) (**c**).

Virus-host interactions were predicted based on shared protein clusters (VC information) with phages from known hosts, shared k-mer frequency patterns, and spacer-protospacer matches. The 34.1 kb and 40.7 kb vOTUs from slick SML were related to *Shewanella* phages based on protein-sharing network analysis (Table S6). These phages and an abundant 106.6 kb vOTU in the FL fraction (463 x coverage) were linked to diverse gammaproteobacterial MAGs based on shared k-mer frequencies, VC information, and spacer to protospacer matches (Fig. 7a, Table S6). Another 57.4 kb vOTU abundant in the virome (185 x coverage) was related to a *Pseudomonas* phage according to vConTACT2 (Table S6) but did not share k-mer frequency patterns with any of the MAGs. Two additional phages of 69.6 and 191.8 kb length detected only in slick SML had conflicting host evidence (Fig. 7a). MAGs from orders Flavobacteriales and Rickettsiales were k-mer linked to minimum 51 and 52 vOTUs, respectively, and likely represent hosts for viruses in the first 70 cm of the water column (Fig. S13).

Four MAGs comprised high-confidence CRISPR arrays (Table S4a), but from these only a 32 bp spacer from the *Paraglaciecola* sp. (MAG_65) CRISPR array matched a protospacer of the highly abundant 106.6 kb vOTU exclusively detected in slick SML. In addition, we found another CRISPR spacer from a *Paraglaciecola* sp. MAG from slick SML matching 104 phages of the genus *Barbavirus* previously isolated on *R. baltica* [53, 104] (Table S9). In addition, the genomes of *P. tunicata* SMS2 and *R. baltica* SMS4 had a CRISPR array, with a spacer of SMS2 matching a 34.8 kb vOTU only detected in the slick SML (Table S9; Supplementary Results).

Among CRISPR spacers recovered from metagenomic reads, we detected 378, 326, 360 and 349 CRISPR spacer-protospacer matches at 100% similarity in slick SML, non-slick SML, slick SSW, and non-slick SSW, respectively. These spacers originated from 29 different CRISPR arrays (Fig. S14). Slick SML-derived spacers targeted protospacers of six out of the nine slick SML specific vOTUs, namely the most abundant 57.4 kb (C1) and 106.6 kb (C3) vOTUs, and the 191.8 kb (C2), 34.1 kb, 40.7 kb (both C4), 69.6 kb (C5) vOTUs (Fig. 7, Table S10). Interaction clusters C1–C4 contained CRISPR spacers originating from different CRISPR arrays/DR sequences (Fig. S14), suggesting infection histories with various bacteria. BLASTing the CRISPR DR sequences from those arrays revealed that bacteria hosting these arrays belonged to different Gammaproteobacteria*,* e.g., spacers from arrays P15 (C3), P16 (C3), P22 (C1), P40 (C1), P43 (C1) (Table S11, Fig. S14). In addition, one DR sequence had a BLAST hit to Flavobacteria (P108, Table S11), and associated CRISPR spacers matching the virus were derived from all four sample types.

### Viral micro-diversity is associated with particles in the upper 70 cm of the water column

Across all samples, 120 viral strain variants were found for 41 different vOTUs (Fig. 7c, Table S12a–i). From those 120 variants, certain singletons were only detected in slick SML (*n* = 16), non-slick SML (5), slick SSW (17), and non-slick SSW (23, Table S12a). The PA fraction had the least number of total variant clusters (62) compared to FL (292) and viral fraction (371). Within all sample comparisons, including water type and filtered fraction, singletons of viral variants were mostly associated with the PA fraction. This shows that the PA microbiome in the upper cms of the water column, including the SML, is related to unique viral variants independent of slick conditions.

### Three phages lytic for *Alishewanella* sp. and *P. tunicata* extracted from slick SML

**Fig. 8:**
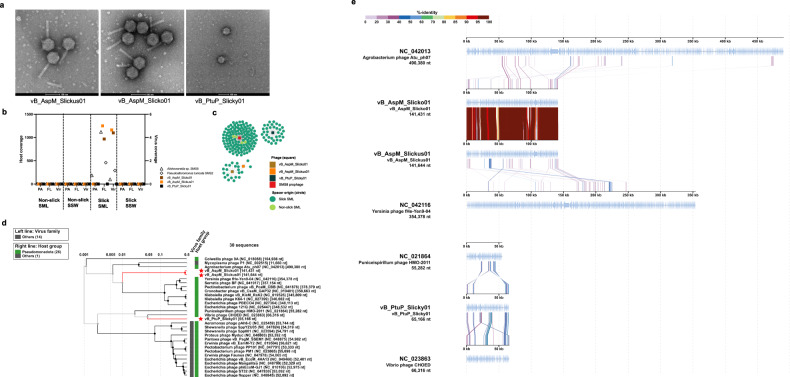
Transmission electron microscopy images, abundance, CRISPR spacer matches and synteny of lytic phage isolates extracted from slick SML. Negatively stained electron micrographs reveal myovirus morphology of Slickus and Slicko and podovirus morphology of Slicky, scale bar 100 nm (**a**). Coverage of reads based on mapping to the host MAGs *Alishewanella* sp. (MAG_01) and *Pseudoalteromonas tunicata* (MAG_66) as well as phages vB_AspM_Slickus01, vB_AspM_Slicko01, and vB_PtuP_Slicky01 (**b**). CRISPR spacer extracted from reads matching at 100% similarity to phage genomes from isolates and the SMS8 prophage of 50 kb. No matches from SSW spacers were detected. **c** Proteomic tree with placement of Slickus, Slicko, and Slicky (indicated by red stars) and closest related phages from the Refseq database (**d**). Based on insights from the tree, genomic alignments with related phages with indicated identity of proteins based on tBLASTX results (**e**). Annotations for the phage isolates are given in Table S14. SML sea-surface microlayer, SSW subsurface water.

Two phages with myovirus morphology and lytic activity against *Alishewanella* SMS8 were isolated from slick SML at sites #1 and #3, and one phage with podovirus morphology for *P. tunicata* from slick SML at site #1 (Fig. 8a). The phages were named *Alishewanella* phage vB_AspM_Slickus01, vB_AspM_Slicko01, and *P. tunicata* phage vB_PtuP_Slicky01 in accordance with Kropinski, Prangishvili and Lavigne [105] (further referred to as Slickus, Slicko, and Slicky). Slickus and Slicko had a mean head diameter of 85.8 ± 2.9 nm (*n* = 11) and 84.0 ± 2.6 nm (*n* = 15), and a mean tail length of 126.6 ± 3.5 nm (*n* = 8) and 126.1 ± 8.1 nm (*n* = 12), respectively. Slicky’s head diameter was 65.2 ± 2.5 nm (*n* = 5) (Table S13). Slicky was distantly related to *Vibrio* phage CHOED and *Puniceispirillum* phage HMO-2011, whereas Slickus and Slicko shared protein identities with *Agrobacterium* phage Atu_ph07 and *Yersinia* phage fHe_Yen9-04 among others (Fig. 8d, e). Due to intergenomic similarity of 91.4% in VIRIDIC for the *Alishewanella* sp. phages, we here propose the new genus “Alishvirus” with species names “Alishvirus slickus” (for strain Slickus) and “Alishvirus slicko” (for strain Slicko). For Slicky, we propose genus and species names “Pseutunvirus” and “Pseutunvirus slicky”, respectively. The genomes of Slickus, Slicko, and Slicky had a length of 141644, 141431, 65166 bp with 195, 198, and 81 open reading frames, respectively, and all carried transfer RNAs. Read mapping showed that Slickus and Slicko were only detected in the slick SML, while Slicky was below detection limit in all metagenomes (Fig. 8b). However, all three phages were targets of CRISPR spacers isolated from slick SML, and the SMS8 prophage additionally by spacers from non-slick SML but not from SSW (Fig. 8c). Functional annotations of Slickus, Slicko, and Slicky are provided in Table S14. To our best knowledge, these are the first phage isolates reported for these host species. Slickus, Slicko, and Slicky tested negative for cross infections in a host range experiment involving other slick SML bacterial isolates (see Supplementary Results).

## Discussion

In this work, we provide new insights on viral-bacterial diversity and interactions in the SML of a natural surface slick from the Baltic Sea—a feature that can form on water surfaces under calm conditions. Metagenomic analysis revealed distinct viral and bacterial communities with specific functional properties in slick SML in comparison to non-slick SML and SSW. The combination of cultivation and sequencing allowed us to identify abundant and important bacteria and phages that we would have missed with metagenomics alone.

Our study distinguishes two types of bacteria and viruses: 1.) “surface cosmopolitans”, i.e. bacteria and viruses that are present in all sample types and might be transferred into the slick with rising bubbles [106], and 2.) “slick-specific opportunists”, i.e. bacteria and viruses being detected in slick SML only and responding quickly to emerging slick conditions. Group 1 comprised a VC from slick SML related to flavobacterial phages as well as the many vOTUs matching *Flavobacteriaceae* and *Rickettsiaceae* MAGs. Flavobacterial phages often occur in temporal proximity to phytoplankton blooms and heterotrophic bacterial communities following up on such blooms [107], for instance in three consecutive summers at the Linnaeus Microbial Observatory in the Baltic Sea [104]. In addition, we found several flavobacterial (*Flaviramulus* sp. MAG_06, *Flavobacterium* sp. MAG_08) and an alphaproteobacterial MAGs (*Rickettsiaceae* MAG_166) in SML and SSW representing potential hosts for these phages. A CRISPR array with DR sequence linked to Flavobacteria had spacers from SML and SSW samples matching different vOTUs, indicating presence of this CRISPR system in both water layers. Common in the Baltic Sea, *Cellulophaga baltica* strains are hosts for diverse flavobacterial phages with varying infection susceptibility [108, 109]. *Cellulophaga* and *Polaribacter* were predicted as actively replicating in the FL fraction of the slick.

Group 2 includes viruses infecting Gammaproteobacteria, and some of those were detected exclusively in the slick SML, presumably because they were beyond detection limit in the other samples. The prevalence of Gammaproteobacteria in short-lived but particle-rich slicks [27] corresponds to a similar occurrence in ephemeral sea foams from the air-water boundary [11] and comparable virus-host ratios (>40) in foams and slicks [33]. During slick formation, which can happen within an hour [110] and some slicks persist only for < 2 h [16], Gammaproteobacteria like *R. baltica*, *Alishewanella* sp., and *P. tunicata* can possibly respond quickly to labile carbon compounds as reported for other Gammaproteobacteria [111, 112]. The prevalence of motility-mediating chemotaxis genes in slick isolates and MAGs suggests that these bacteria actively migrate towards and/or within the slick, possibly responding to higher DOC. Presence of GH103 and GH171 in *R. baltica* suggests the ability to degrade peptidoglycan from detrital bacterial matter, which might be prevalent in the slick due to high cell numbers and viral activity. In addition, *R. baltica* and *Alishewanella* sp. contained homoserine lactone genes, as often found in biofilms [113], indicating that intraspecific communication is probably important in the slick SML. Contrasting CAZyme patterns of low- and high-abundant MAGs in the slick SML suggests that these groups occupy different trophic niches. A similar pattern was observed for the *P. tunicata* and *R. baltica* isolates, potentially supporting their co-existence in the slick. High abundance of *Alishewanella* in the PA fraction indicates a surface-associated lifestyle and biofilm-forming abilities, supported by the observation of biofilms in flask cultures of SMS8 and SMS9 (data not shown). Also *P. tunicata* favors a surface-associated lifestyle in the marine environment [114]. Hence, both taxa have properties that likely facilitate establishment within the SML featuring biofilm-like properties [27].

Gammaproteobacterial hosts from slick SML possess a slick-specific repertoire of CRISPR spacers to interact with slick-specific vOTUs, and also *P. tunicata* SMS2 contained a CRISPR array, a known feature for this species [114]. Remarkably, the same slick vOTU was targeted by spacers from different CRISPR arrays, likely belonging to different host strains due to the conservation of DR sequences [115]. Abundant slick vOTUs seemingly tried to infect multiple hosts within class Gammaproteobacteria, despite such broad host ranges being rare in nature [116, 117]. In line with our observations, densely packed microbial consortia, as found in mats and biofilms, were recently shown to favor viral interactions with hosts of phylogenetically distant microbes, which was reflected by CRISPR spacer-protospacer matches [118]. Protospacers could have also been acquired from defective phages [119] or from intact phages without phage replication being necessarily successful. Potentially, a high viral micro-diversity enables different host ranges, or that the same protospacers exist in different viruses. Extensive spacer exchange might be enhanced by a higher likelihood for virus-host encounters through enhanced virus-host coupling in the neuston compared to the plankton [33].

While viral macro-diversity (Shannon-Wiener index) was lower in slick SML and the PA fraction, we found evidence that viral strain diversification was associated with the PA fraction independent of slick conditions. Particles might constitute substrates for viruses and increase their residence time in given water layer, favoring viral mutations under high-light conditions in the sea surface. Via the “viral shuttle” this would also explain the high level of viral micro-diversity contributing to differential viral abundance patterns on abyssal particles [120], however, future work must be conducted to confirm these dynamics.

The finding of lytic infections in two abundant slick bacteria by three phage isolates suggested that virus-host dynamics in slick SML follow the kill-the-winner theorem [35, 36]. The presence of a prophage in *Alishewanella* sp. SMS8 (Fig. S6) with no similarities to the lytic phages of *Alishewanella* sp. allows speculations that harsh environmental conditions at the air-sea interface occasionally favor the prophage state in abundant and highly proliferating slick bacteria, because we otherwise found only few prophages in the MAGs. Alternatively, lysogeny could be favored when slick disintegrates and host cell numbers decline.

The physicochemical environment at the air-sea interface (high surfactant loads, low surface tension) compared to SSW likely contributed to the distinct viral-bacterial community structures in the slick. *Pseudomonas* and *Pseudoalteromonas* are known producers of surfactant and exopolysaccharides [121], and accordingly, slick-SML dwelling *P. tunicata* encoded genes for biosurfactant production. Surfactants could contribute to lowering surface tension and hence facilitate surface colonization. Furthermore, compounds like surfactin also exert anti-viral properties [122]. Light conditions could additionally shape viral-bacterial communities in slicks considering the varying responses of *Oceanospirillales, Pseudoalteromonas*, and Flavobacteria to light dependent on the particle attachment status [123], but this factor was not considered in this study. The SML is strongly affected by diel light effects [124], and microbes have to adapt to solar and ultraviolet radiation, for instance by pigmentation [3, 125]. This matches the purple-blue coloring of *Alishewanella* SMS9 cultures. Blue color of all *R. baltica* strains and *P. tunicata* SMS2 are likely attributed to glaukothalin and violacein (Table S2f), respectively [114, 126].

## Conclusions

The slick SML harbored distinctive viral-bacterial communities, with genomic and phenotypic features that potentially facilitate colonizing short-lived and dynamic slick habitats. Gammaproteobacteria in slick SML showed functional adaptations such as pigmentation, prevalence of chemotaxis and quorum sensing genes, higher diversity of CAZymes, biofilm formation, as well as adaptive immunity towards specific slick phages. Despite lower diversity in the slick, phages infected typical and abundant slick bacteria. The enrichment of different vOTUs in SML indicates that viral proliferation is likely a strategy to increase the chance for point mutations to circumvent attacks by the host’s adaptive immunity. We conclude that virus-host interactions align with the peculiarity of slicks and are partially uncoupled from underlying waters, despite the connectivity between SML and SSW being generally high, with many shared bacteria and viruses between these two ecosystems [33]. High selective pressure by the “extreme” conditions in the slick, including an active virus-host arms race, might favor discovery of previously unknown viruses and bacteria, although future work needs to study whether the here identified slick vOTUs are indeed unique to this habitat. Viral lytic activity combined with a high DOC level suggest that slicks act as a temporary reservoir of organic material, representing hotspots for the microbial and viral shunt at the air-sea interface constituting a so-far understudied factor for carbon cycling in the sea.

### Supplementary Information

Supplemental Material

Supplementary Tables

## Data Availability

Cell and VLP abundance, surfactant, and DOC data are deposited at PANGAEA, https://doi.pangaea.de/10.1594/PANGAEA.955904 [127]. Raw sequence data, MAGs, bacterial isolate genomes, and the viral metagenome for this project are stored in Bioproject PRJNA855638 at NCBI. Genomes of phage isolates Slickus, Slicko, and Slicky are stored under accessions OQ508956 – OQ508958 at GenBank. For further information on accession numbers, please see Table S15.
